# Effective RNAi-Mediated Silencing of the *Mismatch Repair MSH2* Gene Induces Sterility of Tomato Plants but Not an Increase in Meiotic Recombination

**DOI:** 10.3390/genes12081167

**Published:** 2021-07-29

**Authors:** Svetlana R. Strelnikova, Anastasiya A. Krinitsina, Roman A. Komakhin

**Affiliations:** 1 All-Russia Research Institute of Agricultural Biotechnology, 127550 Moscow, Russia; ankrina@gmail.com (A.A.K.); komakhin@gmail.com (R.A.K.); 2 Biological Faculty, Lomonosov Moscow State University, 119234 Moscow, Russia

**Keywords:** meiosis, crossing over, *MSH2* gene, DNA mismatch repair, recombination, tomato

## Abstract

In plant breeding, the ability to manipulate meiotic recombination aids in the efficient construction of new allelic compositions of chromosomes and facilitates gene transfer from wild relatives of crop plants. The DNA mismatch repair system antagonizes meiotic recombination. In this research, a trial was conducted to evaluate transgenic tomato plants carrying an RNA interference (RNAi) construct designed to inhibit the expression of the mismatch repair *MSH2* gene. To drive the RNAi construct, we used either a pro-SmAMP2 promoter from *Stellaria media* ANTIMICROBIAL PEPTIDE2 or a Cauliflower mosaic virus 35S promoter (CaMV35S). The results of real-time PCR showed that, with a 16 h light/8 h dark photoperiod, MSH2-RNAi tomato transgenic plants exhibited *MSH2* gene transcript contents ranging from 0% to 3% in the leaves, relative to untransformed controls. However, with this lighting mode, the MSH2-RNAi transgenic plants grew slowly, flowered poorly, and did not form seed sets. During cultivation with a 12 h light/12 h dark photoperiod, MSH2-RNAi transgenic plants exhibited *MSH2* gene transcript contents ranging from 3% to 42%, relative to untransformed controls. Under these conditions, F_1_ hybrid seed sets formed for most of the MSH2-RNAi transgenic plants with the RNAi construct driven by the CaMV35S promoter, and for one transformant with the RNAi construct driven by the pro-SmAMP2 promoter. Under conditions of a 12 h light/12 h dark photoperiod, most of the F_1_ transgenic hybrids showed *MSH2* gene transcript contents ranging from 3% to 34% and formed F_2_ offspring sets, which made it possible to assess the meiotic recombination frequency. We showed that the effective inhibition of *MSH2* in MSH2-RNAi tomato transgenic plants is not associated with an increase in meiotic recombination compared to the control, but it stimulates the sterility of plants. It was established that the expression of the *MSH2* gene in tomato plants is about 50 times higher with a 12 h light/12 h dark than with a 16 h light/8 h dark photoperiod. It is discussed that, in *Solanum lycopersicum* tomato plants, which are not sensitive to the day length for flowering, changing the lighting time may be a means of controlling the meiotic recombination frequency within certain limits.

## 1. Introduction

Meiosis is a fundamental process shared by most sexually reproducing eukaryotic organisms. It generates genetic diversity through three principal mechanisms: (i) pairs of homologous chromosomes are independently sorted into haploid gametes; (ii) pairs of homologous chromosomes reciprocally exchange (crossing over) chromosome segments, altering the association of maternal and paternal alleles; and (iii) gene conversion occurs during homologous recombination [1].

In plant breeding, gaining control over the processes which alter crossing-over positions on chromosomes and increase the crossover frequency allow the more efficient construction of new allelic compositions of chromosomes for crop plants [2,3,4]. The ability to manipulate meiotic crossing over may be useful to facilitate the transmission of allelic genes from wild relatives to cultivated plants [5].

In meiosis, crossing over is triggered by the generation of double-strand breaks (DSBs) in DNA throughout the genome, and the repair of these by various biochemical pathways is necessary for the correct completion of meiosis [6]. One of the DSB repair pathways, double-strand break repair, leads to crossing over [7]. During crossing over, regions of heteroduplex DNA containing unpaired bases can locally arise between homologous chromosomes. The mismatch repair (MMR) system eliminates these regions.

The MMR system is a highly conserved pathway that exists in all organisms. The first step of mismatch recognition in eukaryotes is carried out by the homologs of prokaryotic MutS proteins, namely, MSH protein subunits. There are eight homologs of MutS in eukaryotes, MSH1 to MSH8; MSH7 is found only in plants [8] and MSH8 is found in Euglenozoa [9]. MSH proteins recognize mismatches as heterodimers; MutSa (MSH2–MSH6) repairs base–base mismatches or 1–2-nucleotide insertion–deletion loops [10,11], while MutSb (MSH2–MSH3) recognizes larger insertion–deletion loops containing up to 14 nucleotides [12,13]. Plants form an additional heterodimeric complex known as MutSc (MSH2–MSH7) [8], which recognizes some base–base mismatches and reportedly plays a role in meiotic recombination [14]. The *MSH2* gene is one of the key elements of MMR in plants. In meiosis, MMR is capable of destroying heteroduplex DNA and suppressing crossing over [1].

The inactivation of the *MSH2* mismatch repair gene in interspecific yeast hybrids (*Saccharomyces cerevisiae* × *Saccharomyces paradoxus*) increases the frequency of crossing over between homeologous chromosomes and improves spore viability [15]. The knockout of the *MSH2* gene (Atmsh2-1) by T-DNA insertional mutagenesis increases microsatellite instability and somatic recombination in *Arabidopsis thaliana* plants, indicating a decrease in the efficiency of the MMR system in plant cells [16]. In this study, it was shown that the inhibition of MMR by RNA interference (RNAi) targeting of the *MSH2* gene transcript also causes microsatellite instability, but its level varies significantly in different transgenic *Arabidopsis* lines. Another study showed that the Atmsh2-1 mutation stably increases the frequency of meiotic recombination between marker genes against the isogenic background (Landsberg × Landsberg) of *Arabidopsis*, although this effect is weaker against a nonisogenic background (Columbia × Landsberg) [17]. It was recently reported that disruption in the *MSH2* gene by CRISPR-Cas9 in indica rice can create genetic variability [18].

Since no knockout mutants of the *msh2* gene had been found in tomatoes, strategies for the inhibition of the *MSH2* and *MSH7* genes using RNAi and/or dominant-negative constructs were previously used to stimulate meiotic recombination [5]. The production of the AtMSH2-DN2 mutant protein from *Arabidopsis* or inhibition of the *MSH7* gene transcript by RNAi allowed a modest increase in the frequency of meiotic recombination between homEologues of the *S. lycopersicum* tomato hybrid heterozygous for *Solanum lycopersicoides* Dunal chromosome 8. However, at the same time, it was found that the silencing of the *MSH2* gene transcript by RNAi is not an effective way to increase the frequency of meiotic recombination between homEologues. In comparison with Atmsh2-1 knockout mutants of *Arabidopsis* and transgenic tomato plants with overexpression of AtMSH2-DN2, the absence of effects in transgenic tomato plants with *MSH2* gene transcript silencing by RNAi could be attributed to the incomplete suppression of the *MSH2* gene’s expression. This being due to the use in the genetic construct of the well-known Cauliflower mosaic virus 35S promoter (CaMV35S), the effectiveness of which is very limited in the floral organs of plants [19,20]. In addition, it is possible that the RNAi, usually targeting a region of the tomato *MSH2* gene transcript mainly encoding the domain I of the MSH2 protein, tended to be inefficient. It also cannot be ruled out that an insufficient number of transgenic tomato lines with independent transformation events were used to assess the effect of RNAi targeted to the *MSH2* gene transcript on meiotic recombination.

We previously established the nucleotide sequences encoding domains III, IV, and, partially, V of the MSH2 protein in five wild tomato species and the tomato cultivar Marglobe [21]. We found that the differences between these nucleotide sequences of the *MSH2* gene transcripts from different species did not exceed 1%; therefore, even in interspecific tomato hybrids, a single construct based on the conserved region of the tomato *MSH2* gene transcript can be used for silencing by RNAi. In addition, we previously developed a new gene promoter pro-SmAMP2 from *Stellaria media* ANTIMICROBIAL PEPTIDE2, which, in the leaves of transgenic tobacco plants (*Nicotiana tabacum* L.), is 2–3 times stronger than the viral promoter CaMV35S [22,23]. The pro-SmAMP2 promoter is also effective in the floral organs of transgenic tobacco plants, including the stem, sepals, pistil, stigma, anthers, pollen, and the microsporocytes during prophase I of meiosis [24] when crossing over occurs. We hypothesized that a new genetic construct for RNAi that targets alternative regions of the *MSH2* gene transcript and is under the control of the strong and constitutive pro-SmAMP2 promoter, would be more efficient for silencing the tomato *MSH2* gene.

The purpose of this study was to develop an effective technique for *MSH2* gene silencing by RNAi and to assess its effect on meiotic recombination between marker genes in intraspecific tomato hybrids.

## 2. Materials and Methods

### 2.1. Plants for the Experiments

In the present study, plants from the Marglobe tomato line (*S. lycopersicum*) with dominant alleles of the *Wv* and *D* genes located on chromosome 2 were used. To assess the frequency of meiotic recombination, we used plants of the tomato marker line Mo938 with recessive alleles of the *wv* (*white virescent*) and *d* (*dwarf*) genes on chromosome 2, linked at a frequency of about 29% [25]. The plants were cultivated in a greenhouse with a 16 h light/8 h dark photoperiod at 22–24 °C unless otherwise specified. In the light period, the illuminance was 150 µmol/m^2^.

### 2.2. Genetic Constructs for Plant Transformation

When creating genetic plasmid constructs for *Agrobacterium* transformation of plants, the sequence of the *MSH2* gene of the Marglobe tomato line, obtained by us previously [21], was used as a DNA template to obtain the RNAi construct. The analysis of DNA sequences was performed with the use of Basic Local Alignment Search Tool software (BLAST, https://blast.ncbi.nlm.nih.gov/Blast.cgi (accessed on 20 May 2021)).

The RNAi mechanism for silencing the *MSH2* gene transcript was created in several steps. Initially, a 963 bp amplicon was obtained using PCR with the antM2plus and antM2minus primers (Table 1), designated as antM2.

**Figure 1:**
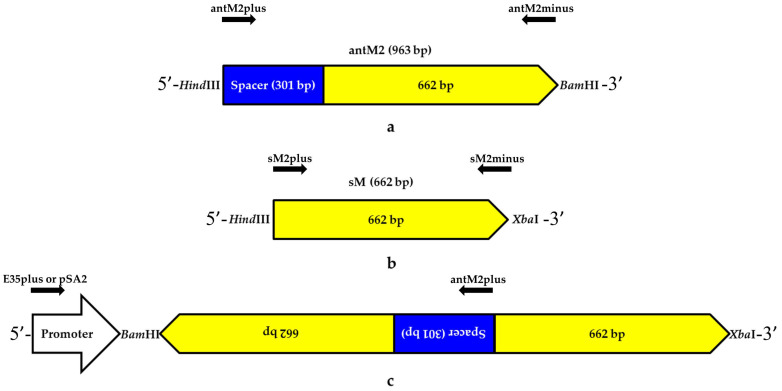
Genetic construct for the RNA interference targeting the *MSH2* gene transcript of tomato plants. (**a**) Scheme of the antM2 nucleotide sequence (963 bp) including the spacer sequence (301 bp); (**b**) scheme of the sM2 nucleotide sequence (662 bp) complementary to 662 bp in the antM2 sequence; (**c**) relative positions of the antM2 and sM2 nucleotide sequences and the gene promoter for their transcription in plant cells. Positions of the primers used for cloning and molecular analysis are shown with the black arrows; primer designations are specified above the arrows.

This amplicon contained a 662 bp region necessary for the formation of an RNA double-stranded structure and a 301 bp spacer, which played the role of a loop in the construct. Due to the sequences of the primers, the restriction sites *HindIII* and *BamHI* were used (Figure 1a).

Then, using the PCR method and the sM2plus and sM2minus primers (Table 1), a 662 bp amplicon partially identical to antM2 and containing *HindIII* and *XbaI* restriction sites was obtained; it was designated sM2 (Figure 1b). Both amplicons were ligated into the pGEM T-easy plasmid to form pGEM-antM2 and pGEM-sM2 plasmids. The *HindIII-BamHI* fragment of pGEM-antM2 was ligated into the previously created plasmid pQE-licBM2-KM2-Mys25 [27] and prehydrolyzed at the same restriction sites to obtain the pQE-antM2 construct. Then, the *HindIII-XbaI* fragment sM2 from pGEM-sM2 was ligated into pQE-antM2, which was previously hydrolyzed at the same restriction sites, to form the plasmid pQE-antM2-sM2, which contained the RNAi construct for silencing *MSH2*. The *BamHI-XbaI* fragment of pQE-antM2-sM2, which has a length of 1625 bp, was ligated into the previously created plant expression vector p35S-recA [28] and pretreated with the same enzymes. As a result, a plasmid for the *Agrobacterium* transformation of plants containing the functional construct antM2-sM2 under the control of the Cauliflower Mosaic Virus 35S promoter was created (CaMV 35S) (Figure 1c); it was designated p35S-antM2-sM2.

The *EcoRI-BglII* fragment of the previously created plasmid p822 containing the pro-SmAMP2 promoter from the *S. media* plant [24] was ligated into pQE-antM2-sM2, which was prehydrolyzed at the *EcoRI* and *BamHI* sites to obtain the plasmid pQE-pro-SmAMP2-antM2-sM2. Next, the *EcoRI-PstI* fragment of pQE-pro-SmAMP2-antM2-sM2 was ligated into the plant expression vector pCambia2300 at the same sites to obtain a plasmid for *Agrobacterium* transformation, in which the antM2-sM2 construct was under the control of a plant-derived pro-SmAMP2 promoter. This plasmid was designated pSmAMP2-antM2-sM2. The vectors p35S-antM2-sM2 and pSmAMP2-antM2-sM2 contained the *neomycin phosphotransferase II (nptII)* gene, allowing for the selection of transformed plant cells in the presence of the antibiotic kanamycin.

### 2.3. Genetic Transformation of the Tomato Plants

The *Agrobacterium* transformation of tomato plants was carried out as described previously [28]. The *Agrobacterium tumefaciens* strain AGL0, carrying the vector p35S-antM2-sM2 or pSmAMP2-antM2-sM2, was used to transform tomato plants.

### 2.4. Analysis of Tomato Plants with Polymerase Chain Reaction

For the detection of *Agrobacterium* contamination, previously developed primers for the *VirE2* gene sequence were used [28]. To detect the hybridization region for the plasmid vectors containing the antM2-sM2 sequence and gene promoters, corresponding primers were used (Table 1). The quantitative content of the mRNA (transcript) of the *MSH2* gene, with normalization relative to the *Actin* gene of tomato, was determined simultaneously by combining a reverse transcription reaction and real-time PCR (“in one tube”). To this end, the following components were added to the reaction mixture: TaqMan probes: ROX (5 (6) -carboxy-X-rhodamine)/BHQ2 for the *Actin* gene and FAM (5 (6) -carboxyfluorescein)/BHQ1 for the *MSH2* gene; primers complementary to the *Actin* gene (Fwd-act and Rev-act) and the cDNA for the *MSH2* gene transcript (msh2+1 and msh2-1); total RNA as a template; and MMLV-RT reverse transcriptase (Syntol, Moscow, Russia). The reaction temperature profile was as follows: 45 °C for 900 s; 95 °C for 300 s; 50 cycles of 95 °C for 15 s and 60 °C for 40 s. The fluorescence level was recorded at the end of each cycle using a CFX96 Touch Real Time System amplifier (Bio-Rad, Hercules, CA, USA). The real-time PCR data were normalized using the 2^−ΔΔCT^ method [29]. The measurements were repeated at least three times using young leaves 3.0 ± 0.5 cm in length.

### 2.5. RNA Extraction

Total RNA was extracted from plant leaves using Trizol reagent (Thermo Fisher Scientific, Waltham, MA, USA) according to the instructions of the manufacturer. To eliminate genomic DNA contamination, the RNA was treated with DNase RQ1 RNase-Free (Promega, Madison, WI, USA) and stored at −70 °C.

### 2.6. Statistical Data Processing

For statistical data processing, Student’s *t*-tests were performed in Microsoft Excel. The mean values and standard deviations are presented. The statistical evaluation of phenotype segregation was carried out using the *chi*-square test (χ^2^) [30]; the recombination frequency (rf) was calculated using the method of maximum likelihood [31].

### 2.7. Pollen Viability

The pollen viability of plants was assessed by acetocarmine staining, as described in [32]. Mature anthers were squashed in 1% acetocarmine in 45% glacial acetic acid, and the percentage of stained pollen grains was determined. For each plant, three anthers were squashed separately, and 100 pollen grains were scored from each anther. The results were averaged across the three anthers.

## 3. Results

### 3.1. Genetic Constructs Created for RNA Interference Targeting the MSH2 Gene in Tomato Plants

In our study on the silencing of *MSH2* by RNAi, we paid close attention to exons 4 and 5 of the *MSH2* gene, which partially encode domain II, completely encode core domain III, and partially encode clamp domain IV of the MSH2 protein. Using the BLAST software, we made sure that this target sequence is unique in the *S. lycopersicum* genome.

### 3.2. Primary Transformants of Tomato Plants Created with a Reduced Content of MSH2 Gene Transcript

The *Agrobacterium* transformation of the tomato Marglobe line was performed using both expression vectors, p35S-antM2-sM2 and pSmAMP2-antM2-sM2. Tomato regenerants arising from independent transformation events were selected in a growth medium containing the antibiotic kanamycin: 22 and 36 plants with the plasmids p35S-antM2-sM2 and pSmAMP2-antM2-sM2, respectively.

**Figure 2:**
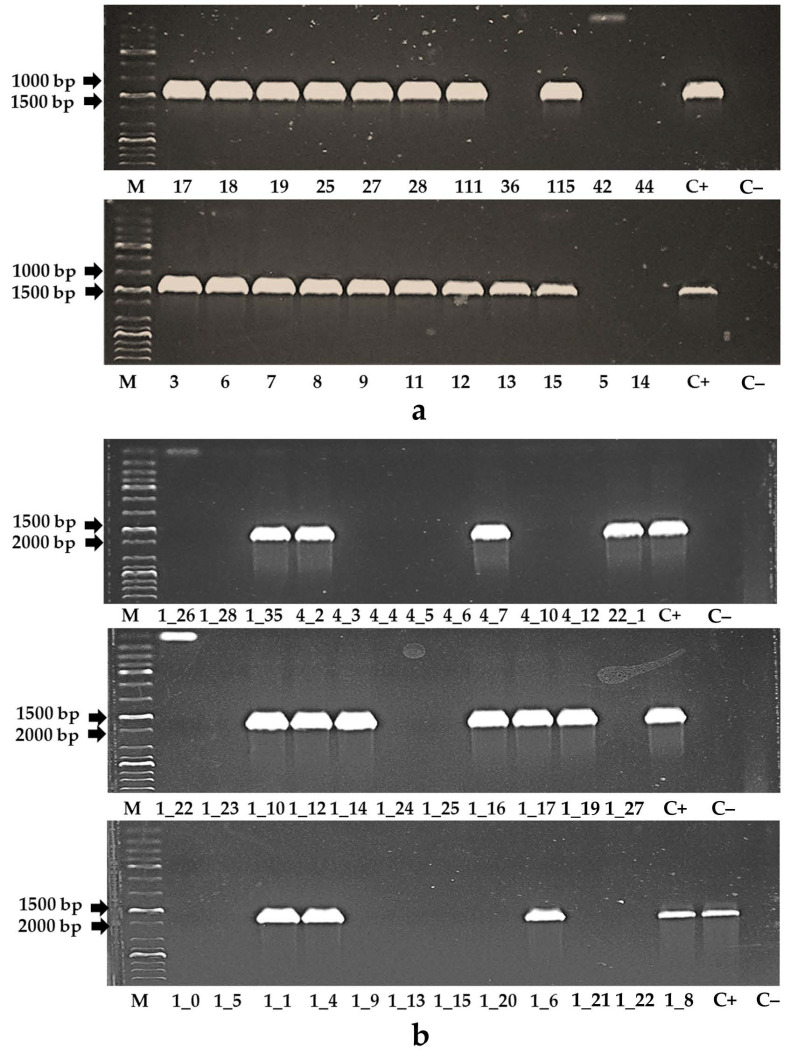
PCR analysis of DNA from tomato regenerants obtained using the plasmid vectors p35S-antM2-sM2 (**a**) and pSmAMP2-antM2-sM2 (**b**). The numbers below the gel images indicate the numbers of the individual transformants. “M” is a GeneRuler 1 kb Plus DNA Ladder (Thermo Fisher Scientific, Waltham, MA, USA). “C+” is a positive control: pSmAMP2-antM2-sM2 (**a**) or p35S-antM2-sM2 (**b**) constructs, respectively, were added as a matrix.

Using PCR and primers for the *VirE2* gene, we confirmed the absence of *Agrobacterium* contamination in all the DNA samples from the leaves of the regenerants. Using the E35plus forward primer for the 5′-end of the CaMV35S promoter, the pSA2 forward primer for the 5′-end of the pro-SmAMP2 promoter, and the antM2plus reverse primer for the 3′-end of the antM2 sequence within the antM2-sM2 construct (Figure 1c), the transformants containing regions of the genetic constructs were identified among the regenerants (Figure 2).

As shown in Figure 2a, out of 22 regenerants, 17 plants were identified as transformants containing recombinant sequences from the p35S-antM2-sM2 construct with an expected length of about 1200–1300 bp. Analogously, 15 transformants with recombinant sequences from the pSmAMP2-antM2-sM2 construct with an expected length of about 1800–1900 bp, were selected from 35 regenerants (Figure 2b). The primary transformants of tomato were designated as T_0_p35S-antM2 and T_0_pSmAMP2-antM2, and these were adapted to greenhouse conditions. However, only 11 of the 17 p35S-antM2-sM2 plants and 13 of the 15 pSmAMP2-antM2-sM2 plants were able to vegetate in the greenhouse.

**Figure 3:**
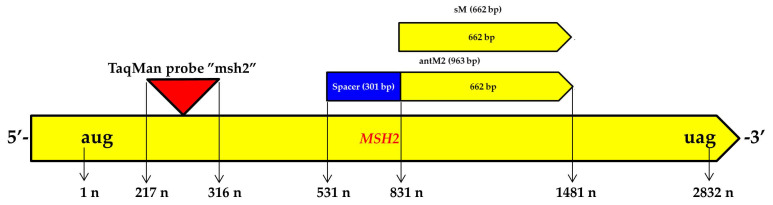
Scheme of the *MSH2* gene transcript and target regions for RNAi and the “msh” TaqMan probe. The first nucleotide of the AUG start codon of the *MSH2* gene transcript’s translated region is designated 1 n; the last nucleotide of the UAG stop codon is designated 2832 n. Arrows and numbers show the relative positions of the antM2 and sM2 sequences, as well as the “msh” TaqMan probe in the *MSH2* gene transcript sequence.

To quantify the expression of the *MSH2* gene by real-time PCR, a TaqMan probe was developed and designated as “msh” (Figure 3).

As shown in Figure 3, the “msh” TaqMan probe targets the *MSH2* gene transcript region, which is located upstream of the target for silencing by RNAi using the antM2-sM2 construct. As a reference for measuring the *MSH2* gene transcript level by real-time PCR (RT-PCR for the measurement of mRNAs), the “housekeeping” *Actin* gene for which the TaqMan probe had been previously developed was used (Table 1).

**Figure 4:**
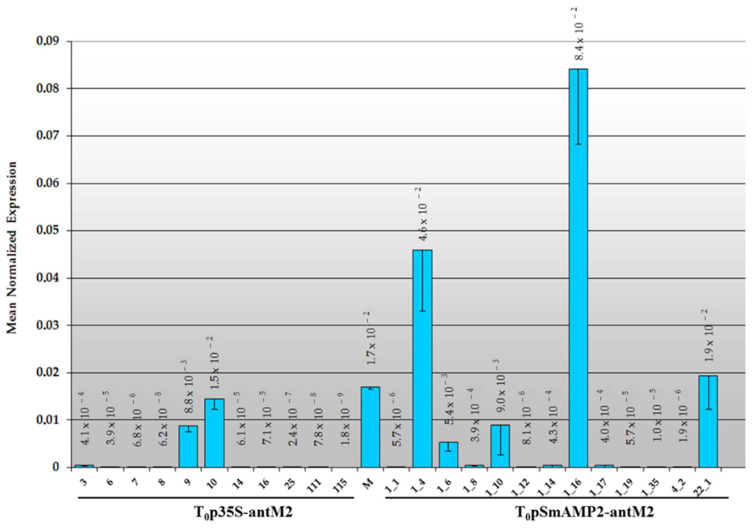
Expression of the *MSH2* gene, normalized relative to that of the *Actin* gene, in tomato leaves under “long-day” (16 h light/8 h dark) conditions based on real-time PCR results. “M” means intact plants from the Marglobe line (control). The numbers below the abscissa axis show the designations of the individual transformants. The vertical lines show the standard errors.

The level of the *MSH2* gene transcript in tomato transformants growing for eight months in a greenhouse under “long-day” (16 h light/8 h dark) conditions was measured four times, and the mean was calculated (Figure 4).

Figure 4 shows that, under “long-day” conditions, the *MSH2* gene transcript level in the young leaves of the intact tomato plants of the Marglobe line was 1.7 ± 0.1 × 10^−2^, about 60 times lower than the expression level of the *Actin* gene. Under the same illumination conditions, the *MSH2* gene transcript level ranged from 1.8 × 10^−9^ to 1.5 × 10^−2^ among individual T_0_p35S-antM2 plants and from 1.9 × 10^−6^ to 8.4 × 10^−2^ among individual T_0_pSmAMP2-antM2 plants.

In some tomato primary transformants (designated as MSH2-RNAi plants), regardless of the genetic construct used for the RNAi, the *MSH2* gene transcript level did not differ significantly from the control (T_0_p35S-antM2 Nos 9 and 10, and T_0_pSmAMP2-antM2 Nos 1_6, 1_10, and 22_1), and in some cases, it exceeded it (T_0_pSmAMP2-antM2 Nos 1_4 and 1_16). Despite this, most of the T_0_pSmAMP2-antM2 (ca. 60%) and T_0_p35S-antM2 (>80%) transformants demonstrated significantly lower *MSH2* gene transcript levels (*p* = 0.05) than the intact Marglobe plants. In general, in these T_0_p35S-antM2 plants, the residual *MSH2* gene transcript content varied from 0% to 3% in comparison with the levels in intact plants. Among the T_0_pSmAMP2-antM2 plants, the value of this indicator ranged from 0% to 14%. For the subsequent study, samples with a residual *MSH2* gene transcript level not exceeding 3% were also selected from T_0_pSmAMP2-antM2 plants.

**Figure 5:**
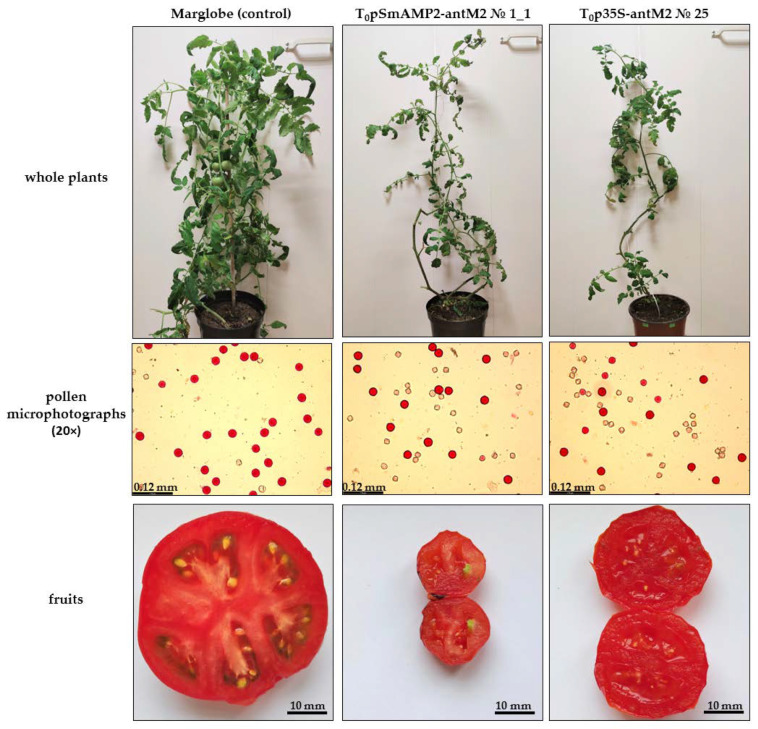
The morphology of several examples of MSH2-RNAi plants under “long-day” (16 h light/8 h dark) conditions.

When MSH2-RNAi tomato plants were growing under “long-day” conditions, we noticed that, compared to the intact Marglobe plants, they grew slowly, flowered poorly, and did not form fruits and seed sets as a result of self-pollination (Figure 5).

This was unexpected, since in the individual MSH2-RNAi plants, the pollen viability according to staining with acetocarmine was at an acceptable level (from 30% to 60%) and viable pollen grains did not differ in size and shape from pollen grains from the intact Marglobe line. Additionally, numerous attempts to pollinate them with pollen from the tomato Mo938 marker line during the year (12–13 months) failed to produce F_1_ hybrids from any of the T_0_pSmAMP2-antM2 transgenic plants. At the same time, the formation of a number of seedless fruits was noted (Figure 5). The pollination of T_0_p35S-antM2 transgenic plants with Mo938 pollen made it possible to obtain fruits and viable seed sets (from 3 to 5 seeds) of F_1_ hybrids only from plants Nos 3 and 16. Intact plants of the Marglobe and Mo938 lines under the same lighting conditions formed fruits with numerous seed sets from self-pollination and cross-pollination.

**Figure 6:**
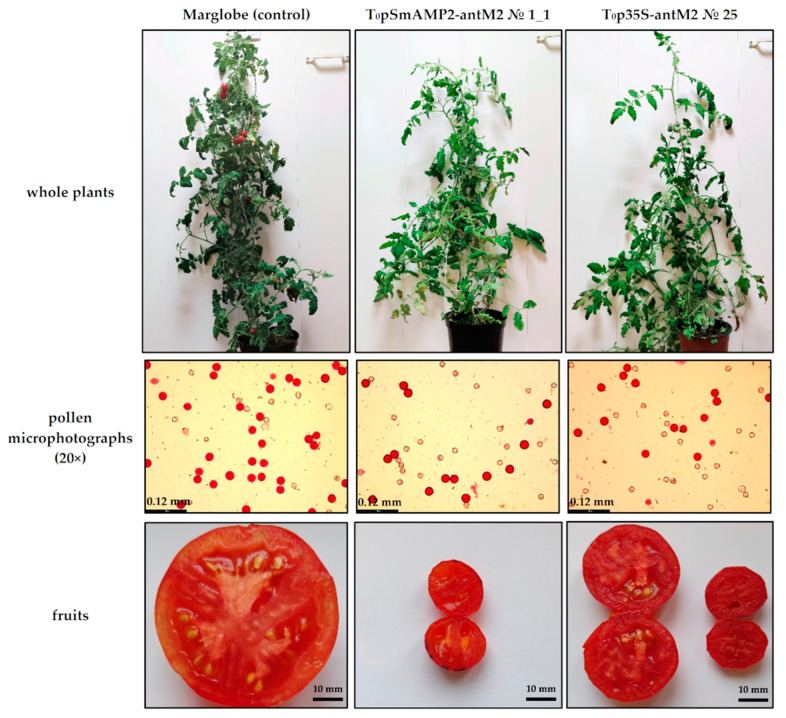
The morphology of several examples of MSH2-RNAi plants under “short-day” (12 h light/12 h dark) conditions.

We changed the lighting regime from “long day” to “short day” (12 h light/12 h dark) and grew the MSH2-RNAi tomato plants for another six months. As a result, some transformants developed fruits from self-pollination and from pollination with pollen from the Mo938 line (Figure 6).

**Figure 7:**
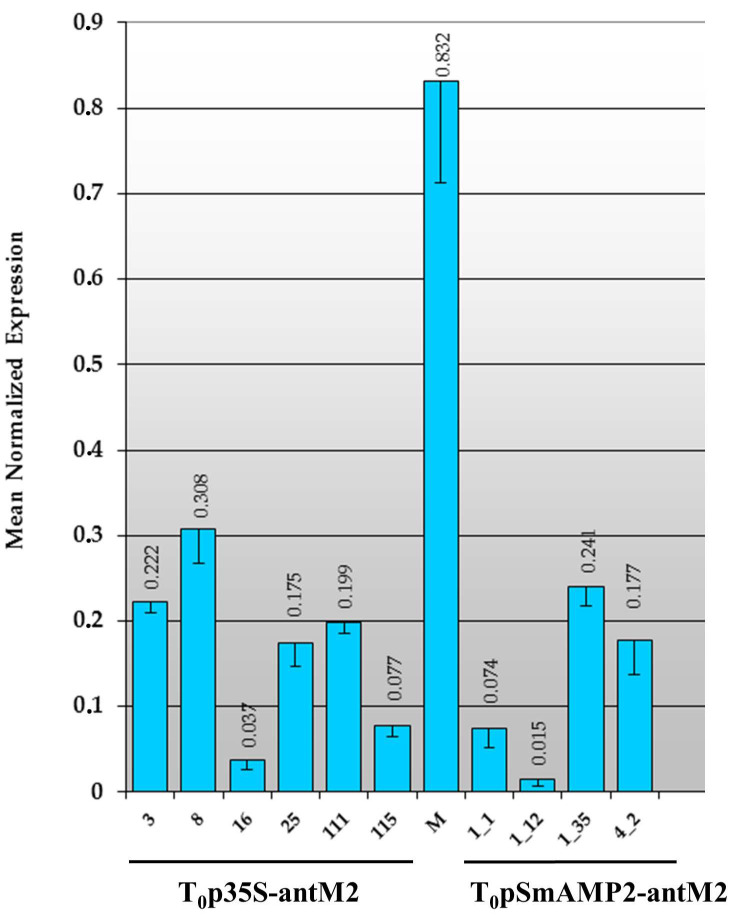
Expression of the *MSH2* gene, normalized relative to that of the *Actin* gene, in tomato leaves under “short-day” conditions (12 h light/12 h dark) based on real-time PCR results. “M” means intact plants of the Marglobe line (control). The numbers below the abscissa axis show the designations of the individual transformants. The vertical lines show the standard errors. The average results for three measurements are presented.

To validate the efficiency of silencing by RNAi under “short-day” conditions, the *MSH2* gene transcript levels were measured (for the first time they were measured three weeks after the lighting regime change from “long day” to “short day”) in MSH2-RNAi plants from both the T_0_p35S-antM2 and T_0_pSmAMP2-antM2 groups, which formed fruits (Figure 7).

Figure 7 shows that the *MSH2* gene transcript level was significantly higher (*p* = 0.05) in all the plants under the “short-day” conditions than under the “long-day” conditions (Figure 4). In intact plants of the Marglobe line, the *MSH2* gene transcript level increased by more than 40 times and reached 8.3 × 10^−1^, while in the MSH2-RNAi plants, it increased by 50 times or more. Despite this, all of the MSH2-RNAi plants demonstrated *MSH2* gene transcript levels that were significantly lower than those in the intact samples. Under “short-day” conditions, the residual expression of the *MSH2* gene in the plant leaves varied from 5% to 42% in the T_0_p35S-antM2 plants and from 3% to 32% in the T_0_pSmAMP2-antM2 plants. Under the “short-day” conditions, the level of the expression of the *MSH2* gene in the leaves of the MSH2-RNAi plants was higher than that in the control plants under the “long-day” conditions (Figure 4 and Figure 7).

For plant growth under “short-day” conditions, it was found that all the T_0_pSmAMP2-antM2 transformants, with the exception of No. 4_2, still did not form seed sets upon self-pollination and upon pollination with pollen from the Mo938 line, although the number of seedless fruits increased. In the case of the T_0_pSmAMP2-antM2 transformant No. 4_2, we succeeded in obtaining several viable F_1_ hybrid seed sets. We also succeeded in obtaining viable F_1_ hybrid seed sets from the pollination of the T_0_p35S-antM2 transformants Nos 8, 25, 111, and 115 with pollen from the Mo938 line. In addition, seed-bearing fruits were formed as a result of self-pollination in the T_0_p35S-antM2 transformants Nos 3, 6, 16, 25, 111, and 115. In the last case, number of seeds in seed sets were from 3% to 10% relative to seed sets of the intact Marglobe plants. Intact plants of the Marglobe and the Mo938 lines formed fruits with numerous seed sets from self-pollination and cross-pollination under the same lighting conditions.

We failed to obtain hybrid seed sets through the pollination of Mo938 plants with the pollen of some transformants from both the T_0_p35S-antM2 and T_0_pSmAMP2-antM2 groups; in this case, the formation of even seedless fruits was not observed.

### 3.3. Assessment of the MSH2 Gene Transcript’s Silencing Efficiency in Tomato Hybrids

**Figure 8:**
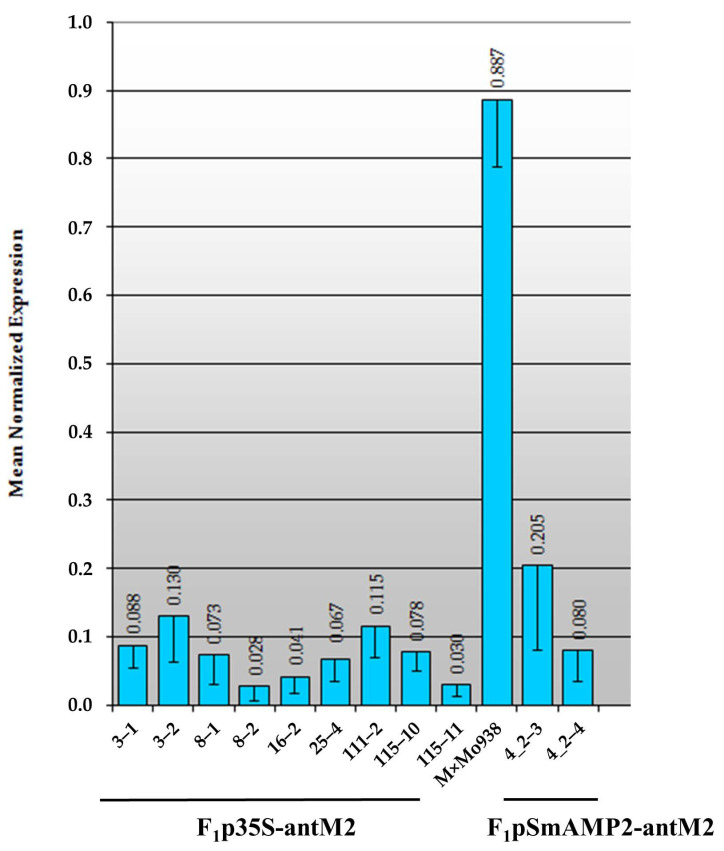
Expression of the *MSH2* gene, normalized relative to that of the *Actin* gene, in the leaves of tomato hybrids under “short-day” conditions (12 h light/12 h dark) according to the results of real-time PCR. MxMo938 are nontransgenic Marglobe × Mo938 hybrids (the average values for two plants are presented). The numbers below the abscissa axis indicate the designation of individual hybrids. The vertical lines show the standard errors. The average results for three measurements over 6 months are presented.

Most of the F_1_ hybrid seeds obtained by the pollination of MSH2-RNAi plants with pollen from the Mo938 line were viable. Among F_1_ hybrids, using PCR and primers for the fusion sequences in the p35S-antM2 and pSmAMP2-antM2 constructs (Figure 1c), we identified the segregation of F_1_ plants into transgenic hybrids, which were designated as F_1_p35S-antM2 or F_1_pSmAMP2-antM2, and nontransgenic F_1_ hybrids. The transgenic hybrids and control (nontransgenic) hybrids were grown under “short-day” conditions to obtain the numerous F_2_ offspring required for the meiotic recombination assay. During the vegetation period, expression of the *MSH2* gene in transgenic hybrids was quantified, while the nontransgenic hybrids were used as comparative controls (Figure 8).

It follows from the data presented in Figure 8 that *MSH2* transcription was significantly lower in the F_1_p35S-antM2 and F_1_pSmAMP2-antM2 plants than in the nontransgenic F_1_ hybrids. On the whole, the *MSH2* transcript level in the transgenic hybrids was comparable to that in the MSH2-RNAi plants under “short-day” conditions (Figure 7). Among all the transgenic F_1_ hybrids, the residual expression of the *MSH2* gene varied from 5% to 34% in relation to the expression level in nontransgenic F_1_ hybrids. By pollen staining with acetocarmine, it was found that, in all the transgenic hybrids, the level of viability varied from 65% to 87%; no significant differences in this parameter between different F_1_ transgenic plants during the growing season were found. In the control hybrids, the level of pollen viability was at least 91%.

As a result of self-pollination, we succeeded in obtaining seed sets of the F_2_ generation from only 7 out of 11 transgenic hybrids from both the F_1_p35S-antM2 and F_1_pSmAMP2-antM2 plants, while only five of them had a sufficient number of offspring to assess the frequency of meiotic recombination using linked marker genes of chromosome 2. In most cases, the transgenic hybrids obtained on the basis of MSH2-RNAi plants capable of forming seed sets during self-pollination turned out to be sufficiently fertile.

### 3.4. Analysis of the Meiotic Recombination Frequency between Marker Genes of Chromosome 2 in Tomato Hybrids with the MSH2 Transcript Silenced

It was found that in the F_2_ progeny from all the F_1_ hybrids, only the monogenic inheritance of genes of the marker loci occurred (Table 2).

Under “short-day” conditions, the meiotic recombination frequency between the *wv* and *d* genes in control F_1_ hybrids was 23.8 ± 1.8% (Table 2), which is lower than the value of 26.7 ± 0.4% found in F_1_ hybrids of the same crossing combination under “long-day” conditions [26]. However, the differences found were not statistically significant (*p* = 0.05).

In individual transgenic hybrids of the F_1_p35S-antM2, the meiotic recombination frequency was within a narrow range, from 20.3% to 23.3%, with an average of 22.3 ± 0.7%, which was indistinguishable from the values of the control F_1_ hybrids in this experiment.

In the F_1_pSmAMP2-antM2 hybrid 4-2-4, the meiotic recombination frequency was 24.6 ± 1.0%, and this did not differ from the control in this experiment.

## 4. Discussion

We included in our research eight and nine independent MSH2-RNAi tomato transgenic plants from T_0_pSmAMP2-antM2 and T_0_p35S-antM2 groups, respectively, with at least 97% RNAi silencing of the *MSH2* transcript. Abnormalities in plant growth, fruit formation, and seed set were found in all MSH2-RNAi plants under “long-day” conditions. However, under “short-day” conditions similar defects (first of all, in fruit and seed set formation) were found in seven out of eight transformants from the T_0_pSmAMP2-antM2 group and only in three out of nine transformants from the T_0_p35S-antM2 group.

It was previously noted that the knockout mutation of the *MSH2* gene in *Arabidopsis* plants leads to the intensive accumulation of mutations in the genome over a number of generations, partial fertility loss, and a small seed set [16,33]. Silencing the *StMSH2* gene with an antisense construct or with a dominant-negative construct with a mutated *AtMSH2* coding sequence led to multiple phenotypic anomalies in somatic potato hybrids [34]. No phenotypic abnormalities in growth and development were initially observed in tomato plants when the *MSH2* and *MSH7* genes were silenced by RNAi [5]. However, the authors of that article reported that 20% of the transformants had ploidy disorders as a result of *Agrobacterium* transformation, and they were not used in further research. Later, in the Arka Vikas tomato cultivar, in response to the RNAi silencing of *MSH2* with an efficiency of more than 80%, defects in the development of flowers and anthers, a low pollen viability level, tetraploid microsporocytes, low fruit productivity, and a reduced seed set were found [35]. However, in this research only one transgenic line of the three studied clearly demonstrated the phenotype described above. In addition, as noted in the Introduction, plants form a heterodimeric complex, MutSc (MSH2–MSH7) [8], which reportedly plays a role in meiotic recombination [14]. It was expected that the disruption of *MSH2* gene function might affect the effectiveness of a heterodimeric (MSH2–MSH7) complex. Therefore, in discussion, we also pay attention to the second component of the heterodimeric complex MutSc, namely, the MSH7 protein. In particular, RNAi for the *MSH7* gene in transgenic barley plants (*Hordeum vulgare* L.) also exhibited a reduced seed set and pollen viability [14]. In wheat plants (*Triticum aestivum* L.), *Tamsh7-3D* mutation also reduces pollen viability but does not affect plant fertility [36]. Thus, disrupted function of the *MSH7* gene leads to similar defects as the disrupted function of *MSH2*, i.e., reduced seed set and pollen viability. Therefore, our results are similar to most of the previously published research indicating a decrease in plant fertility and number of seeds in a seed set as a result of disruptions in the function of the *MSH2* gene. The exact molecular mechanisms leading to the loss of fertility are not clear. Only in the research on the Arka Vikas tomato cultivar with the RNAi silencing of *MSH2,* did the authors attempt to interrelate the decrease of pollen viability and plant fertility with the disruptions in meiosis leading to the reduction of the seed set [35].

In this research, the pollen viability in the MSH2-RNAi tomato plants with at least 97% silencing of the *MSH2* transcript by RNAi under “long-day” conditions ranged from 30% to 60%. The pollen grains from MSH2-RNAi plants did not differ in size and shape from the pollen grains of the intact Marglobe plants (Figure 5 and Figure 6). The pollen viability in the MSH2-RNAi tomato plants was comparable to the pollen viability in the tomato transgenic plants of the Marglobe line with the expression of the *Escherichia coli recA* gene [28]. The transgenic plants expressing the *recA* gene were pollinated with pollen from the Mo938 line and formed numerous fruits and viable seed sets. Similarly, there was no difficulty in crossing tomato transgenic plants of the Marglobe line expressing *SPO11* genes from yeast (*Saccharomyces cerevisae*) and *A. thaliana*, with the Mo938 line [26]. Note that we initially cultivated tomato transgenic plants with simultaneous silencing of the *MSH2* gene and overexpression of *SPO11* genes under “long-day” conditions. This is comparable to the results of a previously published study involving Arka Vikas MSH2-RNAi tomato lines, of which only one line with high silencing efficiency showed about 10% pollen viability, while the rest of the lines with lower silencing efficiency had a significantly higher pollen viability level [35]. However, our further results demonstrated that in MSH2-RNAi tomato plants, an acceptable level of the pollen viability is not always combined with the formation of fruits and seed sets.

In our study, the MSH2-RNAi tomato plants with at least 97% silencing of the *MSH2* transcript by RNAi under “long-day” conditions, did not form seed sets after self-pollination, despite an acceptable level of pollen viability. It was previously shown that, in the Arka Vikas MSH2-RNAi tomato line with silencing (>80%) of *MSH2* by RNAi, low pollen viability (10%) was associated with the formation of 5–10% seeds [35]. In contrast, in the *Arabidopsis MSH2* mutant lines the seed set on average was reduced to only 50% [33]. In our study, only two T_0_p35S-antM2 transformant plants (Nos. 3 and 16) under “long-day” conditions were able to form from 3 to 5 seeds of F_1_ hybrids after pollination with Mo938 pollen; the rest did not form seeds. However, under “short-day” conditions seven more MSH2-RNAi plants with at least 60% silencing of the *MSH2* transcript by RNAi were able to form F_1_ hybrids seed sets. These results imply that in MSH2-RNAi tomato plants, illumination time affects the effectiveness of disruptions in the function of the *MSH2*.

In our study, we found, for the first time, that the expression of the *MSH2* gene in the leaves of tomato plants was significantly lower when the duration of illumination was increased (Figure 4 and Figure 7). Previously published studies do not mention the effect of the duration of illumination on the level of the *MSH2* gene transcript in tomato plants [5,35]. Our observation itself may explain the greater efficiency of *MSH2* gene transcript silencing by RNAi in MSH2-RNAi tomato plants under “long-day” conditions. In addition, we previously showed that the “long-day” regime increased the efficiency of the pro-SmAMP2 promoter in transgenic tobacco plants in comparison with the “short-day” regime [24]. Consequently, effective *MSH2* gene transcript silencing by RNAi in T_0_pSmAMP2-antM2 tomato lines was a result of a combination of these two factors. There are conflicting data regarding the efficiency of the CaMV35S promoter depending on the duration of illumination. On the one hand, it was shown that CaMV35S-directed expression was not affected by the various light conditions used [37,38,39,40,41]. On the other hand, some authors showed that light suppressed gene expression driven by CaMV35S [42,43,44]. This may be due to the improved stability of *uidA* mRNA in the dark [45,46]. One previously published study clearly showed that, when using the CaMV35S promoter to drive the *uidA* gene, the GUS activity in lettuce plants (*Lactuca sativa* L.) under a 16 h light/8 h dark photoperiod was significantly higher than that under 12 h light/12 h dark and 8 h light/16 h dark photoperiods [47]. Another study showed that the CaMV35S promoter loses its effectiveness in dark conditions in the cells of moss plants (*Physcomitrella patens*) [48]. Since the results of our studies show that the efficiency of *MSH2* gene transcript silencing by RNAi with the CaMV35S promoter is higher in MSH2-RNAi tomato plants with a “long-day” regime, this may indicate a higher efficiency of this promoter with an increase in illumination time.

In our study, the acceptable pollen viability level and the low seed productivity of the MSH2-RNAi tomato plants when pollinated with Mo938 line pollen may also indicate defects of oocytes or failure in embryo development. Initially, in the MSH2-RNAi tomato lines, in response to the RNAi silencing of *MSH2*, the low fruit productivity and seed set were asserted to interrelate with the tetraploid microsporocytes and, as the consequence, with a pollen viability level [35]. However, the authors of this paper did not find ploidy abnormalities among the progeny of the MSH2-RNAi tomato plants and proposed that the absence of triploid progeny may also result from the existence of a strong triploid block in members of Solanaceae, including tomatoes [49,50]. In our research, the absence of ploidy abnormalities among the transgenic and control F_1_ hybrids confirms the monogenic inheritance of marker genes of chromosome 2 (Table 2). However, this does not completely rule out some probability of tetraploid microsporocytes formation in MSH2-RNAi tomato plants. Besides that, in mammalian cells, the *MSH2* gene has been identified as a target of E2F transcription factors [51] wherein the loss of *MSH2* function thus inhibits E2F signaling. It is believed that disruption of E2F signaling affects the progression of the cell cycle and promotes failure of cytokinesis. As the *MSH2* gene has a probable role in the mammalian cell cycle, it is supposed plausible that *MSH2* may affect the cell cycle in plants in a similar fashion [35]. It was shown that the Cd-induced G2/M phase arrest was markedly diminished in *MSH2*-deficient *Arabidopsis* roots [52]. Furthermore, Cd elicited endoreplication in *MSH2*-deficient roots. The results suggest that *MSH2* components of the MMR system are involved in the G2 phase arrest and endoreplication in *Arabidopsis* roots. These results do not rule out that in our research the low fertility of MSH2-RNAi tomato plants might also be related to disruptions in the G2/M phase during pollination, impregnation or embryo development. In our research, the formation of a number of seedless fruits in MSH2-RNAi tomato plants resulting from pollination with the Mo938 line pollen, may be seen as evidence of the above supposition.

The similar phenotypic abnormalities identified in MSH2-RNAi tomato plants in our research and in a previously published study [35] may also be due to the selection of the same target regions in the *MSH2* gene transcript sequence for silencing by RNAi. Previously, the sequence from 416 to 717 n (relative to the AUG start codon) of the translated region of the *MSH2* mRNA was used for silencing by RNAi [35]. In our research, the main target for RNAi was the sequence from 831 to 1481 n and, in addition, the region from 531 to 831 n, which plays the role of a spacer in the antM2-sM2 construct for RNAi, could also participate in the suppression of *MSH2* gene expression as an antisense construct (Figure 3). Thus, in both tomato studies, the *MSH2* region between 531 and 717 n was subject to silencing. Therefore, it cannot be ruled out that the high efficiency of *MSH2* gene silencing and its phenotypic consequences depend on the *MSH2* gene transcript region used as a target for RNAi. Notably, in the first study published on *MSH2* silencing by RNAi, which did not mention phenotypic abnormalities in tomato hybrids, the target was a sequence from 55 to 437 n in the *MSH2* mRNA [5]. At the same time, in the latter study, the limited number of MSH2-RNAi lines, the use of the viral CaMV35S promoter, and the lack of quantitative determination of *MSH2* gene expression could have hindered the involvement of MSH2-RNAi lines with highly efficient RNAi silencing in the studies. Though we did not find in the *S. lycopersicum* genome other targets for the antM2-sM2 construct for RNAi, the defects in MSH2-RNAi plant growth may evidence their existence. Alternatively, one can suppose a more significant role of the *MSH2* gene in the course of vegetative development of the tomato plants.

In our research, the plant-derived pro-SmAMP2 promoter and the virus-derived CaMV35 promoter were comparable in terms of the effectiveness of *MSH2* silencing in tomato leaves under two different lighting conditions (Figure 4, Figure 7 and Figure 8). However, the use of the plant-derived promoter further reduced the ability of MSH2-RNAi plants to form fruits and seed sets. The inability to obtain any progeny from MSH2-RNAi plants with the pro-SmAMP2 promoter in this study may indicate a higher efficiency for the silencing of *MSH2* in floral organs and their constituent tissues compared to when the CaMV35S promoter is used. Although under the “short-day” conditions, the level of the expression of the *MSH2* gene in the leaves of the MSH2-RNAi plants was higher than that in the control plants under the “long-day” conditions (Figure 4 and Figure 7), most T_0_pSmAMP2-antM2 plants remained sterile. The higher efficiency of the pro-SmAMP2 promoter is a consequence of a stable pattern of transgene expression under its control, which encompasses all of the organs studied in transgenic plants, including floral organs, microsporocytes, and pollen [24]. We also previously found that the high expression level of the *uidA* reporter gene driven by the pro-SmAMP2 promoter is comparable in the leaves and anthers of transgenic tobacco plants and is only two times lower in pollen (unpublished data). The ability of one MSH2-RNAi plant (No. 4_2) from the T_0_pSmAMP2-antM2 group to form seed sets can be explained by the integration of T-DNA into the genome region, which limits the efficiency of the pro-SmAMP2 promoter under “short-day” conditions in floral organs but not in leaves. Unlike the pro-SmAMP2 promoter, the CaMV35S viral promoter is significantly less efficient in the floral organs of plants [19]. In particular, the expression pattern of the *uidA* reporter gene driven by the CaMV35S promoter did not include pollen in 43% of the transformants, and in the remaining 57%, the reporter level was 1–2 orders of magnitude lower than that in the leaves. The observed pattern of the CaMV35S viral promoter’s expression in the floral organs of the transgenic plants explain why, in this study, only three out of nine T_0_p35S-antM2 tomato plants showed phenotypic abnormalities in fruit and seed set development, while the other six out of nine T_0_p35S-antM2 tomato plants formed the expected seed set, and their hybrids had an acceptable meiotic recombination level (Table 2). It is probable that, in the MSH2-RNAi plants without phenotypic abnormalities and their F_1_ hybrids, the efficiency of the CaMV35S promoter in the floral organs was zero or low.

In this research, the meiotic recombination in control F_1_ hybrids under “short-day” conditions was lower (Table 2) than that seen previously in similar control F_1_ hybrids under “long-day” conditions [26]. In the context of a decrease in the expression of the *MSH2* gene under “long-day” conditions, these results do not contradict the initial assumption that a decrease in *MSH2* expression has a positive effect on meiotic recombination. Perhaps, in tomato plants that are not sensitive to the day length for flowering, changing the lighting time may be a trigger influencing meiotic recombination within certain limits.

In our study, under “short-day” conditions we obtained an insignificant decrease in the meiotic recombination between the marker genes *wv* and *d* of chromosome 2 in F_1_ hybrids with *MSH2* silencing when using the CaMV35S promoter, relative to the meiotic recombination in the nontransgenic control (Table 2). Previously, in tomato hybrids, when the *MSH2* gene was silenced by RNAi using the CaMV35S viral promoter, slight decreases in the genetic distances between markers were also observed [5]. The similarity of the results obtained in the two studies may be due to the fact that only plants with low RNAi efficiency in the floral organs were used to estimate the frequency of meiotic recombination. In the previous study, for the assessment of genetic distances, the authors selected MSH2-RNAi tomato lines that did not have phenotypic abnormalities and, thus, probably excluded transgenic plants with high *MSH2* gene silencing efficiency from their studies [5]. In our study, the selection of plants with low RNAi efficiency occurred naturally when using the CaMV35S promoter to assess meiotic recombination, since the MSH2-RNAi tomato plants with high *MSH2* silencing efficiency were sterile. The lack of effects in one hybrid, No. 4_2-2, from the T_0_pSmAMP2-antM2 group, as mentioned above, may have been caused by a decrease in the efficiency of the pSmAMP2 promoter due to the integration of T-DNA into a specific region of the tomato genome.

Altogether, these results indicate that, in tomato plants, *MSH2* silencing by RNAi to increase the meiotic recombination frequency has certain limitations. There is probably a certain level of *MSH2* gene expression that is critical for the viability of tomato plants. This may be the reason why *MSH2* gene knockout mutants were not found among the various tomato species.

## Figures and Tables

**Table 1:** Primers for the creation of genetic constructs and molecular analysis of transgenic plants.

Target Sequence	Primer	5′-3′	Amplicon, bp
*MSH2*	antM2plus	aagctttgatagccacttcaccaatt	963
	antM2minus	ggatccgtttgtttgtgcaaattatg	
	sM2plus	aagcttagcaattatggaaactacacagtc	662
	sM2minus	tctagagtttgtttgtgcaaattatgaatc	
	TaqMan-msh	FAMttctcttggagaggatggatcgBHQ1	-
	msh2 + 1	tgcgctttccagtgttagtg	109 *
	msh2-1	tgaaccactgccctcataca	
CaMV35S	E35plus	aacaaagggtaatatccggaaacc	-
pro-SmAMP2	pSA2	acggaattcgtaccaacgtgagtaactat	-
*Actin* (U60482)	TaqMan-act	[26]	65 *
	Fwd-act		
	Rev-act		

* The specificity of the amplification using the primer pairs was checked by electrophoresis in a 1.5% agarose gel, taking the formation of one amplicon as the criterion.

**Table 2:** Frequency of meiotic recombination (mean ± SD) between marker genes *wv* and *d* of chromosome 2 in tomato F_1_ hybrids with *MSH2* silencing by RNAi under “short-day” conditions.

F_1_ Hybrid	F_2_ Phenotypic Classes				χ^2^ (3:1)		rf, %
	WvD	wvD	Wvd	wvd			
					Wv:wv	D:d	
F_1_p35S-antM2 No. 3-1	260	43	43	64	0.26	0.26	23.3 ± 0.2
F_1_p35S-antM2 No. 16-1	465	65	58	104	0.12	0.93	20.3 ± 2.6
F_1_p35S-antM2 No. 111-2	261	40	35	58	0	0.41	22.5 ± 2.2
F_1_p35S-antM2 No. 115-11	513	74	79	104	1.46	0.63	23.0 ± 0.9
Mean F_1_p35S-antM2	1499	222	215	330	1.09	0.49	22.3 ± 0.7
F_1_pSmAMP2-antM2 No. 4-2-4	536	85	93	114	0.41	0	24.6 ± 1.0
F_1_ (Marglobe × Mo938)	393	65	55	72	0.1	3.3	23.8 ± 1.8

For *p* ≤ 0.05 and df = 1, the critical value of χ^2^ is 3.84.

## Data Availability

The data presented in this study are available on request from the corresponding author.

## References

[1] Cole F., Keeney S., Jasin M. (2012). Preaching about the Converted: How Meiotic Gene Conversion Influences Genomic Diversity. Ann. N. Y. Acad. Sci..

[2] De Muyt A., Pereira L., Vezon D., Chelysheva L., Gendrot G., Chambon A., Lainé-Choinard S., Pelletier G., Mercier R., Nogué F. (2009). A High Throughput Genetic Screen Identifies New Early Meiotic Recombination Functions in *Arabidopsis thaliana*. PLoS Genet..

[3] Wijnker E., de Jong H. (2008). Managing Meiotic Recombination in Plant Breeding. Trends Plant Sci..

[4] Lambing C., Franklin F.C.H., Wang C.-J.R. (2017). Understanding and Manipulating Meiotic Recombination in Plants. Plant Physiol..

[5] Tam S.M., Hays J.B., Chetelat R.T. (2011). Effects of Suppressing the DNA Mismatch Repair System on Homeologous Recombination in Tomato. Theor. Appl. Genet..

[6] Mézard C., Tagliaro Jahns M., Grelon M. (2015). Where to Cross? New Insights into the Location of Meiotic Crossovers. Trends Genet..

[7] Page S.L., Hawley R.S. (2003). Chromosome Choreography: The Meiotic Ballet. Science.

[8] Culligan K.M., Hays J.B. (2000). Arabidopsis MutS Homologs—AtMSH2, AtMSH3, AtMSH6, and a Novel AtMSH7—Form Three Distinct Protein Heterodimers with Different Specificities for Mismatched DNA. Plant Cell.

[9] Sachadyn P. (2010). Conservation and Diversity of MutS Proteins. Mutat. Res./Fundam. Mol. Mech. Mutagen..

[10] Acharya S., Wilson T., Gradia S., Kane M.F., Guerrette S., Marsischky G.T., Kolodner R., Fishel R. (1996). HMSH2 Forms Specific Mispair-Binding Complexes with HMSH3 and HMSH6. Proc. Natl. Acad. Sci. USA.

[11] Genschel J., Littman S.J., Drummond J.T., Modrich P. (1998). Isolation of MutSβ from Human Cells and Comparison of the Mismatch Repair Specificities of MutSβ and MutSα. J. Biol. Chem..

[12] Marti T.M., Kunz C., Fleck O. (2002). DNA Mismatch Repair and Mutation Avoidance Pathways. J. Cell. Physiol..

[13] Modrich P. (1991). Mechanisms and Biological Effects of Mismatch Repair. Annu. Rev. Genet..

[14] Lloyd A.H., Milligan A.S., Langridge P., Able J.A. (2007). *TaMSH7*: A Cereal Mismatch Repair Gene That Affects Fertility in Transgenic Barley (*Hordeum vulgare* L.). BMC Plant Biol..

[15] Hunter N., Chambers S.R., Louis E.J., Borts R.H. (1996). The mismatch repair system contributes to meiotic sterility in an interspecific yeast hybrid. EMBO J..

[16] Leonard J.M., Bollmann S.R., Hays J.B. (2003). Reduction of Stability of Arabidopsis Genomic and Transgenic DNA-Repeat Sequences (Microsatellites) by Inactivation of *AtMSH2* Mismatch-Repair Function. Plant Physiol..

[17] Emmanuel E., Yehuda E., Melamed-Bessudo C., Avivi-Ragolsky N., Levy A.A. (2006). The Role of *AtMSH2* in Homologous Recombination in *Arabidopsis thaliana*. EMBO Rep..

[18] Karthika V., Chandrashekar B.K., Kiranmai K., Ag S., Makarla U., Ramu V.S. (2021). Disruption in the DNA Mismatch Repair Gene *MSH2* by CRISPR-*Cas9* in Indica Rice Can Create Genetic Variability. J. Agric. Food Chem..

[19] Wilkinson J.E., Twell D., Lindsey K. (1997). Activities of CaMV 35S and Nos Promoters in Pollen: Implications for Field Release of Transgenic Plants. J. Exp. Bot..

[20] Sunilkumar G., Mohr L., Lopata-Finch E., Emani C., Rathore K.S. (2002). Developmental and Tissue-Specific Expression of CaMV 35S Promoter in Cotton as Revealed by GFP. Plant Mol. Biol..

[21] Krinitsina A., Komakhin R. (2019). Cloning of the Mismatch Repair *MSH2* Gene Fragment from Various Tomato Species. Biomics.

[22] Madzharova N.V., Kazakova K.A., Strelnikova S.R., Snycheva O.A., Vetchinkina E.M., Efremova L.N., Vysotskii D.A., Babakov A.V., Komakhin R.A. (2018). Promoters Pro-SmAMP1 and pro-SmAMP2 from Wild Plant *Stellaria media* for the Biotechnology of Dicotyledons. Russ. J. Plant Physiol..

[23] Efremova L.N., Strelnikova S.R., Gazizova G.R., Minkina E.A., Komakhin R.A. (2020). A Synthetic Strong and Constitutive Promoter Derived from the *Stellaria media* Pro-SmAMP1 and pro-SmAMP2 Promoters for Effective Transgene Expression in Plants. Genes.

[24] Komakhin R.A., Vysotskii D.A., Shukurov R.R., Voblikova V.D., Komakhina V.V., Strelnikova S.R., Vetchinkina E.M., Babakov A.V. (2016). Novel Strong Promoter of Antimicrobial Peptides Gene Pro-SmAMP2 from Chickweed (*Stellaria media*). BMC Biotechnol..

[25] Komakhin R.A., Strelnikova S.R., Zhuchenko A.A. (2019). Genetic Features of the Tomato Marker Line Mo938. Russ. J. Genet..

[26] Komakhina V.V., Krinitsina A.A., Milyukova N.A., Komakhin R.A. (2020). Expression of Recombinant *SPO11* Genes Locally Alters Crossing over in Tomato. Russ. J. Genet..

[27] Komakhin R.A., Abdeeva I.A., Salehi Dzhuzani G.R., Goldenkova I.V., Zhuchenko A.A. (2005). Thermostable Lichenase as a Translational Reporter. Russ. J. Genet..

[28] Komakhin R.A., Komakhina V.V., Milyukova N.A., Goldenkova-Pavlova I.V., Fadina O.A., Zhuchenko A.A. (2010). Transgenic Tomato Plants Expressing *RecA* and *NLS-RecA-LicBM3* Genes as a Model for Studying Meiotic Recombination. Russ. J. Genet..

[29] Muller P.Y., Miserez A.R., Dobbie Z. (2002). Processing of Gene Expression Data Generated by Quantitative Real-Time RT-PCR. BioTechniques.

[30] Suzuki D.T., Griffiths A.J.F. (1976). Introduction to Genetic Analysis.

[31] Kosambi D.D., Ramaswamy R. (2016). The Estimation of Map Distances from Recombination Values. Selected Works in Mathematics and Statistics.

[32] Chetelat R.T. (2016). Overcoming Sterility and Unilateral Incompatibility of *Solanum lycopersicum* × *S. sitiens* Hybrids. Euphytica.

[33] Hoffman P.D., Leonard J.M., Lindberg G.E., Bollmann S.R., Hays J.B. (2004). Rapid Accumulation of Mutations during Seed-to-Seed Propagation of Mismatch-Repair-Defective Arabidopsis. Genes Dev..

[34] Rakosy-Tican E., Lörincz-Besenyei E., Molnár I., Thieme R., Hartung F., Sprink T., Antonova O., Famelaer I., Angenon G., Aurori A. (2019). New Phenotypes of Potato Co-Induced by Mismatch Repair Deficiency and Somatic Hybridization. Front. Plant Sci..

[35] Sarma S., Pandey A.K., Sharma K., Ravi M., Sreelakshmi Y., Sharma R. (2018). MutS-Homolog2 Silencing Generates Tetraploid Meiocytes in Tomato (*Solanum lycopersicum*). Plant Direct.

[36] Serra H., Svačina R., Baumann U., Whitford R., Sutton T., Bartoš J., Sourdille P. (2021). Ph2 encodes the mismatch repair protein MSH7-3D that inhibits wheat homoeologous recombination. Nat. Commun..

[37] Frohnmeyer H., Hahlbrock K., Schafer E. (1994). A Light-Responsive in Vitro Transcription System from Evacuolated Parsley Protoplasts. Plant J..

[38] Cooker D.E., Webb K.J. (1997). Stability of CaMV 35S-*Gus* Gene Expression in (Bird’s Foot Trefoil) Hairy Root Cultures under Different Growth Conditions. Plant Cell Tissue Organ Cult..

[39] Yamamoto Y.Y., Kondo Y., Kato A., Tsuji H., Obokata J. (1997). Light-Responsive Elements of the Tobacco PSI-D Gene Are Located Both Upstream and within the Transcribed Region. Plant J..

[40] Kurata H., Takemura T., Furusaki S., Kado C.I. (1998). Light-Controlled Expression of a Foreign Gene Using the Chalcone Synthase Promoter in Tobacco BY-2 Cells. J. Ferment. Bioeng..

[41] Zambre M., Terryn N., De Clercq J., De Buck S., Dillen W., Van Montagu M., Van Der Straeten D., Angenon G. (2003). Light Strongly Promotes Gene Transfer from *Agrobacterium tumefaciens* to Plant Cells. Planta.

[42] Ellis D.D., McCabe D., Russell D., Martinell B., McCown B.H. (1991). Expression of Inducible Angiosperm Promoters in a Gymnosperm, *Picea Glauca* (White Spruce). Plant Mol. Biol..

[43] Schnurr J.A., Guerra D.J. (2000). The CaMV-35S Promoter Is Sensitive to Shortened Photoperiod in Transgenic Tobacco. Plant Cell Rep..

[44] Zuker A., Ahroni A., Tzfira T., Ben-Meir H., Vainstein A. (1999). Wounding by Bombardment Yields Highly Efficient Agrobacterium-Mediated Transformation of Carnation (*Dianthus caryophyllus* L.). Mol. Breed..

[45] Bovy A., Van Den Berg C., De Vrieze G., Thompson W.F., Weisbeek P., Smeekens S. (1995). Light-Regulated Expression of the *Arabidopsis thaliana Ferredoxin* Gene Requires Sequences Upstream and Downstream of the Transcription Initiation Site. Plant Mol. Biol..

[46] Dickey L., Gallo-Meagher M., Thompson W. (1992). Light Regulatory Sequences are Located within the 5′ Portion of the Fed-1 Message Sequence. EMBO J..

[47] Okayama T., Furukawa H., Okamura K., Murase H. (2010). The Effect of Photoperiod on *β-Glucuronidase* gene Expression under Control CaMV-35S Promoter in Transgenic Lettuce. Environ. Control. Biol..

[48] Saidi Y., Schaefer D.G., Goloubinoff P., Zrÿd J.-P., Finka A. (2009). The CaMV 35S Promoter Has a Weak Expression Activity in Dark Grown Tissues of Moss *Physcomitrella patens*. Plant Signal. Behav..

[49] Ehlenfeldt M.K., Ortiz R. (1995). Evidence on the Nature and Origins of Endosperm Dosage Requirements in Solanum and Other Angiosperm Genera. Sex. Plant Reprod..

[50] Nilsson E. (1950). Some experiments with tetraploid tomatoes. Hereditas.

[51] Ren B., Cam H., Takahashi Y., Volkert T., Terragni J., Young R.A., Dynlacht B.D. (2002). E2F integrates cell cycle progression with DNA repair, replication, and G2/M checkpoints. Genes Dev..

[52] Cao X., Wang H., Zhuang D., Zhu H., Du Y., Cheng Z., Cui W., Rogers H.J., Zhang Q., Jia C. (2018). Roles of MSH2 and MSH6 in cadmium-induced G2/M checkpoint arrest in Arabidopsis roots. Chemosphere.

